# Facial Mimicry in 6–7 Year Old Children with Disruptive Behavior Disorder and ADHD

**DOI:** 10.1371/journal.pone.0084965

**Published:** 2014-01-09

**Authors:** Peter Deschamps, Nicolette Munsters, Leon Kenemans, Dennis Schutter, Walter Matthys

**Affiliations:** 1 Department of Psychiatry, Brain Center Rudolf Magnus, University Medical Center Utrecht, Utrecht, The Netherlands; 2 Department of Experimental Psychology, Helmholtz Research Institute, Utrecht University, Utrecht, The Netherlands; 3 Department of Child and Adolescent Studies, Utrecht University, Utrecht, The Netherlands; University of Reading, United Kingdom

## Abstract

**Background:**

Impairments in facial mimicry are considered a proxy for deficits in affective empathy and have been demonstrated in 10 year old children and in adolescents with disruptive behavior disorder (DBD). However, it is not known whether these impairments are already present at an earlier age. Emotional deficits have also been shown in children with attention-deficit/hyperactivity disorder (ADHD).

**Aims:**

To examine facial mimicry in younger, 6–7 year old children with DBD and with ADHD.

**Methods:**

Electromyographic (EMG) activity in response to emotional facial expressions was recorded in 47 children with DBD, 18 children with ADHD and 35 healthy developing children.

**Results:**

All groups displayed significant facial mimicry to the emotional expressions of other children. No group differences between children with DBD, children with ADHD and healthy developing children were found. In addition, no differences in facial mimicry were found between the clinical group (i.e., all children with a diagnosis) and the typically developing group in an analysis with ADHD symptoms as a covariate, and no differences were found between the clinical children and the typically developing children with DBD symptoms as a covariate.

**Conclusion:**

Facial mimicry in children with DBD and ADHD throughout the first primary school years was unimpaired, in line with studies on empathy using other paradigms.

## Introduction

Empathy is the ability to share and understand the emotions of other people with whom we interact and plays an important role in the development of prosocial behavior and inhibition of antisocial and aggressive behavior [1], [2]. It is assumed that empathy is initiated by the observation of another's emotional state, followed by a cascade of phenomena [3] that have been studied on an emotional (sharing another's emotional state), cognitive (understanding another's emotional state) and behavioral level (e.g., targeted helping) [4]. Although the precise mechanism, how mimicry is related to the development of individual differences in empathy, remains unclear [3], adequate responses to the emotional states of others also involve the activation of corresponding facial, vocal or postural expressions, called mimicry. Previous facial mimicry studies in school-aged children (mean age 10 years) and adolescents (mean age 13 years) with disruptive behavior disorder (DBD) suggest deficits in response to negative but not positive emotions [5]–[7].

Several important issues concerning facial mimicry responses in children with DBD need further exploration. First, it remains unclear how early in development abnormalities in responses to emotional expressions start to emerge. The empathic ability of aggressive children may become increasingly impaired as social demands in peer interactions rapidly increase. Hence, deficits in facial mimicry might already be present in children with DBD at the start of school age (6–7 years old). On the other hand, studies using paradigms other than facial electromyography (facial EMG) (e.g., behavioral observation) suggest that aggressive preschoolers do not differ from their healthy developing peers in their response to the emotions of others [8], [9]. The primary goal of the present study was to determine whether 6 to 7 year old children with DBD already show facial mimicry impairment. Second, despite high co-morbidity of attention-deficit/hyperactivity disorder (ADHD) and DBD and high co-occurrence of ADHD symptoms in children with DBD and DBD symptoms in children with ADHD, little attention has been paid to the influence of ADHD on emotion perception and processing in children with DBD [10], [11]. Several studies in children with ADHD have shown that emotion processing might also be impaired, to some extent, in boys with ADHD [12]–[17]. Interestingly, it has been argued that deficits in responding to the emotions of others in children with ADHD are at least partially accounted for by the co-existence of DBD [15] and that in boys with DBD, deficits might at least partially be related to ADHD [18].

The present study aimed to address these issues by examining facial mimicry responses to emotional facial expressions in a sample of 6–7 year old children with DBD, in children with ADHD, and in healthy developing children. Two lines of approach were followed. First, three groups were compared, i.e., children with DBD, children with ADHD, and typically developing children. Second, while comparing the clinical group (i.e., all children with a diagnosis) to the typically developing group, first the effect of DBD on facial mimicry was examined with ADHD symptoms as a covariate, and second the effect of ADHD was examined with DBD symptoms as a covariate.

## Methods

### Participants

A sample of 100 children ranging from six to seven years old with a previous clinical diagnosis of DBD (i.e., either oppositional defiant disorder (ODD) or conduct disorder (CD)) and/or ADHD was recruited at the Outpatient Clinic of the Department of Child and Adolescent Psychiatry, University Medical Center Utrecht. Children were excluded from participating if a clinical diagnosis of ADHD or DBD was not confirmed (n = 3) in the Diagnostic Interview Schedule for Children (DISC module E) [19] or when they had an estimated IQ below 70 (n = 8) based on the vocabulary and block design subsets of the Wechsler Intelligence Scale for Children III-Dutch version [20], [21]. Eighteen children were excluded as they had taken methylphenidate (n = 18) on the day of testing, despite instructions to cease medication prior to assessment. Furthermore, in six children from the clinical groups no EMG data were collected, either caused by technical difficulties, lack of cooperation or anxiety in the children. The final patient group for analyses comprised 65 children.

The healthy developing control group consisted of 37 children from regular elementary schools in the vicinity of Utrecht who did not meet criteria for a clinical diagnosis of ADHD or DBD on the DISC and had an estimated IQ within the normal range. No EMG data were collected in three children from the control group due to technical difficulties or anxiety. The Medical Ethics Committee of the University Medical Centre Utrecht approved the study protocol and parents gave written informed consent prior to participation.

### Measurements

The DISC module E interview [19] was used to distinguish patient groups. For our first categorical approach, we pooled children with DBD with ADHD (n = 41) and children with DBD without comorbid ADHD (n = 6) in one DBD group. The other patient group consisted of children with ADHD without a comorbid DBD diagnosis (n = 18). Because of the small sample size of the DBD-only group, an analysis comparing this group to other groups was not appropriate. The group of children with DBD (n = 47) included both children with ODD (n = 41) and those with CD (n = 6). For our second approach, a total patient group was analyzed including 65 children with a diagnosis of DBD (n = 6), ADHD (n = 18) or DBD with comorbid ADHD (n = 41).

The Child Behavior Checklist 6–18 (CBCL) and Teacher Report Form (TRF) [22] were collected and used to quantify attention problems and rule-breaking/aggressive behavior.

Table 1 shows the characteristics of the sample used for final data analyses, divided in the DBD group with and without comorbid ADHD, ADHD only group and healthy control group. Analyses presented in Table 1 show that children in the DBD group were on average 4 months younger than the TD children. Furthermore children in the DBD and ADHD groups contained fewer girls, and these children had lower estimated IQ and lower socio-economic status (SES) than children in the control group. Children in the DBD group did not differ from children in the ADHD group in sex, estimated IQ or SES, but were significantly younger. As expected, the three groups significantly differed on attention problems and rule-breaking/aggressive behavior.

**Table 1:** Descriptives.

Characteristics	TD	ADHD	DBD	*F* (df = 96)	Contrasts
	(*n* = 34)	(*n* = 18)	(*n* = 47)		
	*M* (*SD*)	*M* (*SD*)	*M* (*SD*)		
Age	7.1 (0.5)	7.1 (0.7)	6.7 (0.5)	6.90*	TD, ADHD > DBD
Sex: male/female	17/17	8/10	11/36	6.65*	TD ≠ ADHD, DBD
estimated IQ	110 (20)	103 (17)	100 (19)	3.20*	TD > ADHD, DBD
SES	7.0 (2.1)	5.1 (1.9)	5.6 (1.5)	8.27*	TD > ADHD, DBD
CBCL T score					
-Attention	52.7 (4.0)	67.0 (8.4)	66.8 (7.9)	47.07*	TD < ADHD, DBD
-Rule-breaking	53.0 (4.3)	58.0 (6.8)	61.9 (6.4)	22.73*	TD < ADHD < DBD
-Aggression	53.5 (5.6)	63.4 (8.9)	70.8 (7.8)	55.34*	TD < ADHD < DBD
TRF T score					
-Attention	52.0 (3.1)	59.9 (9.7)	61.6 (7.5)	19.71*	TD< ADHD, DBD
-Rule-breaking	50.9 (2.6)	54.9 (5.4)	58.8 (7.7)	16.66*	TD < ADHD < DBD
-Aggression	52.2 (3.7)	60.8 (5.6)	64.5 (10.8)	22.03*	TD< ADHD, DBD

Note: * p<0.05.

### Facial EMG data collection

**Figure 1:**
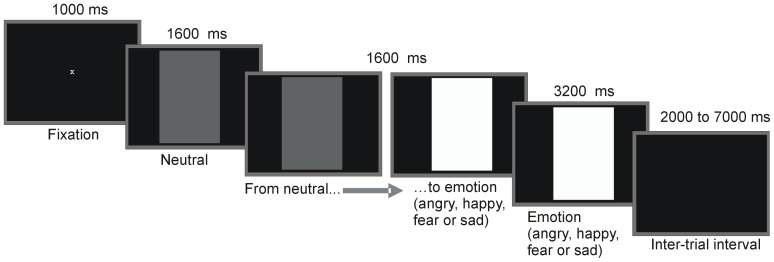
Example trial of the passive viewing task. Each trial started with a central fixation cross, followed by a film clip. The clips started with a neutral expression (in the figure represented by a gray rectangle), followed by a morph into a dynamic emotional expression and ended with a still of the full-blown emotion (in the figure represented by a white rectangle). Each trial ended with an inter-stimulus interval.

Film clips with dynamic emotional facial expressions, created at our laboratory, were used in the present study [23]. In these film clips, each with a total duration of 6400 ms, five different children (two boys and three girls) expressed anger, sadness, fear and happiness as illustrated in Figure 1. Clips started with a 1600 ms static of a neutral expression which served as baseline, followed by a 1600 ms morph into a dynamic emotional expression and ended with a 3200 ms static of the full-blown emotion. Each film clip was preceded by an inter-stimulus interval (a black screen), followed by a central fixation cross with a duration of 1000 ms. In total 32 movie clips were presented, once in a semi-random sequence in a first block (16 clips, 4 children × 4 emotions), and once in a semi-random sequence in a second block (16 clips, 4 children × 4 emotions). The size of the pictures was 21.5 cm height by 16 cm width. They were viewed from a distance of 95 cm. Furthermore, during the task, there were four trials in which a cartoon character was presented during an emotional film clip. Children were instructed to push a response button when the character appeared on screen in order to maintain the child's attention to the faces. The data collected during these trials and during the four familiarization trials were excluded from further analyses.

EMG activity was recorded from bipolar montages from the corrugator supercilii (corrugator), zygomaticus major (zygomaticus), frontalis medialis (frontalis) and depressor anguli oris (depressor), according to the guidelines given by Fridlund and Cacioppo [24]. Ag-AgCl electrodes with a diameter of 4 mm, filled with conductive electrode gel (Signa gel, Parker Laboratories, Inc., Fairfield, New Jersey, U.S.A.), were placed on the left side of the face to obtain maximal reactions [25]. Raw EMG recordings were made with the ActiveTwo system (BioSemi, Amsterdam, The Netherlands) relative to the common mode sense (CMS). The ground consisted of the active CMS and passive driven right leg (DRL) electrode placed on the forehead that form a feedback loop driving the subject's average potential as close as possible to the analog-to-digital converter (i.e., the amplifier “zero”) reference voltage in the A/D-box. The EMG signal was sampled at 2048 Hz.

### Procedure

EMG data were collected while the child was seated in a chair in front of a computer screen in a dimly lit room at their own school. To ensure participants were at ease, they first had a small talk with the experimenter and completed the two WISC-III subtests. Children were instructed to watch the film clips carefully and to push a button when a popular cartoon character appeared. They were told they would receive a small present as a reward upon finishing the task. Between the two blocks of the passive viewing task, the experimenter ensured that the child was both comfortable and motivated. Additionally, during the task an experimenter encouraged the children to pay attention and recorded the time segments when the child was not looking at the computer screen to provide a measure of visual inattention. Total duration of the facial EMG task was approximately 12 minutes.

### Data reduction and analysis

EMG signals were filtered offline (high-pass 20 Hz, 48dB/octave) and full wave rectified using Brain Vision Analyzer Software (Brain Products GmbH, Munich). Trials marked by the experimenter during the task indicating that the child was not looking at the computer screen, were excluded from further analysis. The average number of trials removed per participant was 1.82 (SD 0.41) out of 32 trials in the typically developing group, 3.68 (SD 0.60) in the DBD with/without ADHD group and 4.95 (SD 1.16) in the ADHD only group.

Raw EMG data were segmented into 100 ms epochs. All values were expressed as a percentage of individual baseline activity, defined as the mean activity during 1600 ms neutral facial expression preceding onset of the morph. Averaged activity during the interval starting 500 ms after the beginning of the morphed dynamic expression and ending 500 ms after the beginning of the static expression at the end of the morphed clip was used for further analyses (total time 1600 ms). Mean EMG responses across this 1600 ms period, expressed as a percentage change from baseline activity, were calculated for each emotion-muscle combination (averages of all stimuli for that emotion-muscle combination in the two blocks). Data points that exceeded 3 SD above or below the grand mean change score of the emotion condition were marked as outliers and excluded from further analysis [26]. Mean EMG responses as expressed in percentage change from baseline activity were calculated for each emotion-muscle combination (averages of all responses for that emotion-muscle combination in the two blocks).

Based on previous research of our group [23], facial EMG composite scores were calculated on basis of the absolute mimicry response to all four emotional presentations. Since mimicry to happy facial expressions consists of both smiling activity (i.e., increase in zygomaticus muscle) and relaxation of frowning activity (i.e., decrease in corrugator muscle), to calculate the total mimicry response to happy facial expressions (HAPPY), we used the following formula: [happy mimicry =  (% change in zygomaticus activation during happy stimulus presentation compared to neutral face baseline - % change in corrugator activation during happy stimulus presentation compared to neutral face baseline)/2]. Thus, we calculated the overall mean of the positive change in zygomaticus and the negative change in corrugator activity in response to happy facial expressions compared to neutral face baseline. Likewise, angry facial mimicry consists of an increase in frowning and a decrease in smiling activity, the total angry score (ANGRY) consisted of the overall mean of the positive change in corrugator and the negative change in zygomaticus activity in response to angry facial expressions (formula: [angry mimicry =  (% change in corrugator activation during stimulus presentation compared to baseline- % change in zygomaticus presentation during stimulus presentation compared to baseline)/2)]. The total fear score (FEAR) consisted of the positive change of frontalis activity in response to fearful facial expressions, and the total sad score (SAD) consisted of the positive change in frontalis, corrugator and depressor activity in response to sad facial expressions (formula: [sad mimicry =  (% change in frontalis + % change in corrugator + % change in depressor compared to neutral face baseline)/3]).

Statistical analyses were performed using PASW Statistics 18.0 (IBM Company, Chicago, Illinois). Initially, we validated the composite scores within the healthy control group, as this group was not identical to the group used in our previous study [23]. Using one-sample t-tests, we checked whether the separate muscles of the composite scores changed significantly during presentation of the emotional film clips, compared to the activity during the neutral face baseline.

First, a multivariate analysis of variance (MANOVA) was conducted to examine whether facial mimicry differed in children with DBD, children with ADHD only, and healthy controls. Dependent variables were the facial mimicry response composite scores to sad, fearful, angry and happy facial expressions (SAD, FEAR, ANGRY and HAPPY MIMICRY). MIMICRY was entered as a within subjects factor with two levels (baseline and activation during stimulus presentation). GROUP was entered as between subjects variable with three levels (DBD with or without ADHD, ADHD and healthy controls).

Second, multivariate analyses of variance (MANOVA) were conducted to compare the facial mimicry response scores (SAD, FEAR, ANGRY and HAPPY MIMICRY) in the overall patient group with the typically developing children (GROUP) with the parent and teacher reported attention and aggression symptom scores entered as covariates.

In all tests, the alpha level of significance was set at *p*<0.05 (two-tailed).

## Results

The independent sample t-tests within the healthy control group showed that all four composite scores consisted of the hypothesized muscle activation patterns (all p-values <0.05). In particular, in line with predictions, the presentation of angry facial expressions showed a significant increase in corrugator activity compared to the pre-stimulus neutral face baseline (t(33) = 3.03, p = 0.005) and a significant decrease in zygomaticus activity (t(33) = −2.31, p = 0.027). Following presentation of happy facial expressions, children showed an expected significant decrease in corrugator activity (t(33) = −3.98, p<0.001) and a significant increase in zygomaticus activity (t(33) = 3.41, p = 0.002). Presentation of fearful facial expressions led to an increase in frontalis activity compared to baseline (t(33) = 4.64, p<0.001). Sad facial expressions induced a significant increase in corrugator (t(33) = 4.57, p<0.001), frontalis (t(33) = 4.45, p<0.001) and depressor (t(33) = 2.21, p = 0.034) activity compared to baseline.

A significant main effect of MIMICRY was found, demonstrating that overall, the presented stimuli resulted in facial mimicry (F(4,93)  = 21.49, p<0.001).

Univariate analyses showed a significant effect of MIMICRY in response to SAD (F(1,96) = 33.90, p<0.001), FEAR (F(1,96) = 27.89, p<0.001), ANGRY (F(1,96) = 46.45, p<0.001) and HAPPY (F(1,96) = 32.00, p<0.001) facial expressions.

**Figure 2:**
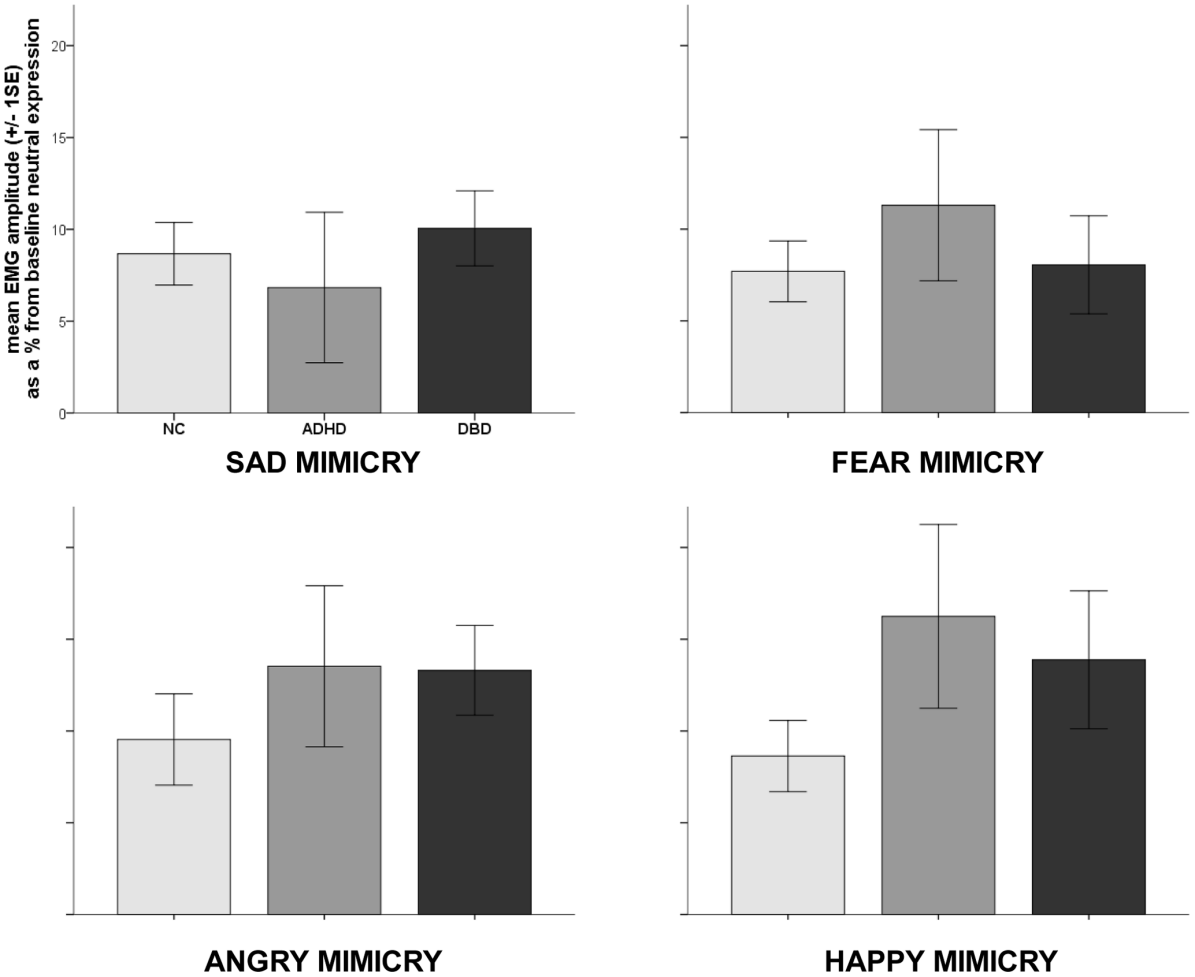
Facial mimicry response to emotional facial expressions in DBD, ADHD and healthy controls. No significant differences were shown between groups in mean EMG amplitude as a percentage from baseline neutral expression for SAD, FEAR, ANGRY and HAPPY MIMICRY presented for healthy controls, children with attention-deficit/hyperactivity disorder (ADHD) and children with disruptive behavior disorder (DBD). Error bars represent +/− 1 standard error.

We did not find a significant multivariate main effect of GROUP (F(8,188) = 0.80, p = 0.60), indicating no differences in facial mimicry between clinical groups and healthy developing children (see Figure 2).

An additional MANOVA comparing the activation of the individual muscles (i.e., zygomaticus and corrugator in response to happy and angry expressions, frontalis in response to fear and corrugator, frontalis and depressor in response to sad) between the three groups showed no multivariate effect of group (F(16,180) = 0.740, p = 0.75) meaning that the absence of a group effect in the main analysis was not due to the use of composite scores.

Next, a second additional analysis was conducted within the boys to assure the imbalance of sex in our groups could not explain the lack of a group difference. This analyses yielded similar results as the main analysis and showed no main effect of group (F(8,116) = 0.92, p>0.50).

Finally, four analyses were conducted to examine the effect of GROUP (all patients versus typically developing children) on facial mimicry with attention and aggression symptom scores as covariates respectively, reported by either parents (CBCL attention and CBCL aggression t scores) or teachers (TRF attention and TRF aggression t scores). No significant multivariate effect of GROUP was found in any of the MANOVAs with these individual factors entered as covariate (all p>0.15). Of note, no significant correlations were found between facial mimicry and the CBCL Attention t score (F(4,93) = 0.87, p = 0.48), CBCL Aggression t score (F(4,93) = 1.40, p = 0.24), TRF Attention t score (F(4,91) = 0.62, p = 0.65), TRF Aggression t score (F(4,91) = 1.11, p = 0.36).

## Discussion

In the present study no evidence was found for impaired facial mimicry in 6–7 year old children with ADHD as compared to healthy controls. Also, no differences were found in facial mimicry between children with DBD and healthy controls. However, since the group of children with DBD without ADHD in our study sample was not sufficiently large, we had to pool the children with DBD with and without comorbid ADHD. Nevertheless, no differences in facial mimicry were found between the clinical group (i.e., all children with a diagnosis) and the typically developing group in an analysis with ADHD symptoms as a covariate, and no differences were found between the clinical children and the typically developing children with DBD symptoms as a covariate.

Results of an absence of facial mimicry deficits in our sample of 6–7 year olds with DBD are in keeping with studies using other paradigms (e.g., behavioral observation) that suggest that aggressive school-aged children and adolescents [2], [27] but not younger children and preschoolers [8], [9] respond less to the emotions of others compared to their healthy developing peers. Since in 10 year old children and adolescents with DBD diminished facial EMG responses have been demonstrated [5]–[7], one may speculate that EMG responses to emotional facial expressions are still intact in 6–7 year old children and that decreases in mimicry responses start after the beginning of school age.

However, there are other possible explanations why we did not find a group difference. Children in our study were younger than those in previous studies that showed facial mimicry deficits in DBD [5]–[7]. Since throughout development into late childhood and adolescence, symptoms of DBD are known to persist in certain, and decline in other children [28], [29], our sample might have included children with less severe psychopathology. The symptom scores on the CBCL filled in by parents and the TRF in the present study indeed were lower as compared to those in previous studies [5]–[7]. Also, children in our study were recruited from an outpatient population, whereas in previous studies children were recruited from inpatient and day-treatment settings [5], [6] or special schools for adolescents with severe behavioral problems [7]. Importantly, the present study sample contained only a few children with CD and the others were diagnosed with ODD, whereas in other studies twenty percent [5], [6] to almost half of the DBD sample consisted of CD children [7]. Recently, it has been suggested that the neurobiology of ODD may be different from CD [30], [31] as ODD differs from CD in symptomatology, comorbidity and development [32]–[35]. Overall, this points towards less severe and different psychopathology in our young outpatient group as a possible explanation for the lack of a group difference.

Facial mimicry in children with ADHD thus far had not been studied, but previous studies using other paradigms had suggested deficits in emotion processing in children with ADHD. Several studies in children with attention problems and ADHD have shown that their facial emotion recognition skills [12]–[14] and empathic responsiveness to emotions [15]–[17] tend to be less well developed compared to healthy children. However, we could not show deficits in facial mimicry in ADHD compared to typically developing children.

With regard to the role of sex differences, our study sample differed from previous studies on facial mimicry in children with DBD as those studies did not examine girls. Little is known about the influence of sex on the development of facial mimicry, but studies in adults have suggested females might show more facial mimicry, although only in response to happy facial expressions [36], [37]. To further examine whether the sex ratio in our study influenced the main findings, we conducted an additional analysis within the group of boys in our study. This analysis showed that, as in the overall sample, boys with DBD or ADHD showed no deficits in facial mimicry. Hence it is unlikely that the presence of girls in our sample influenced our main finding. However, it should be noted that due to the small sample sizes in the subgroup analyses, these analyses were likely to be statistically underpowered to detect this effect.

Finally, there are several methodological differences in our study compared to previous work to consider. First, only one other study examined facial EMG responses to child stimuli [6]. While stimuli of adults are useful to study emotional responsiveness in adult-child interactions, they might provide only limited information on social interactions between children. Next, the procedure and analysis in the present study was developed to maximize attention paid to the stimuli. Namely, children were encouraged to pay attention, motivated with the promise of a reward, an instruction was inserted in the paradigm to catch a cartoon character, and trials marked with visual inattention were excluded from further analysis. This could have reduced the influence of attention problems on deficits in facial mimicry. Two other studies found evidence for a positive moderating influence of increased attention on emotion processing in adults with low empathy and antisocial behavior using a fear-potentiated startle paradigm [38] and in children using a fear recognition task [39]. Both studies suggest that deficits in emotion processing can be at least temporarily corrected by instructing subjects to focus on the eyes of other people and guiding their attention towards relevant parts of the presented stimuli. Until future studies assess facial mimicry simultaneously with objective procedures, like eye-tracking, to verify actual attendance to the stimuli, it remains difficult to unravel whether previous findings of impaired mimicry are partly driven by a lack of attention. Further study is needed to explore whether young children with DBD and/or ADHD are only capable to adequately make use of their mimicry system under optimal conditions, i.e., conditions that need not be ecologically valid. It might well be that in children with ADHD a continuous lack of proper attention to relevant parts of emotional facial stimuli in daily live has a negative effect on the development of emotion processing and recognition.

Since in 10 year old children and adolescents with DBD diminished facial EMG responses have been demonstrated [5]–[7], one may speculate that EMG responses to emotional facial expressions are still intact in 6–7 year old children and decreases in mimicry responses start after the beginning of school age. Longitudinal studies using facial EMG and other physiological assessment methods are needed to shed light on the development of responsiveness to visual and other sensory modalities of emotional stimuli of other children. Further study should identify whether, at what age, and in which subgroups (e.g. those with CD versus those with ODD) children with DBD become impaired in their responding to emotions, and which factors affect altered emotional responsiveness. In conclusion, this study demonstrates that 6–7 year old children with DBD and ADHD exhibit normal facial mimicry to emotional facial expressions.

## References

[1] Eisenberg N, Miller PA (1987) The relation of empathy to prosocial and related behaviors. Psychol Bull 101: 91–119. 3562705

[2] Miller PA, Eisenberg N (1988) The relation of empathy to aggressive and externalizing/antisocial behavior. Psychol Bull 103: 324–344. 3289071 10.1037/0033-2909.103.3.324

[3] Hofelich AJ, Preston SD (2012) The meaning in empathy: distinguishing conceptual encoding from facial mimicry, trait empathy, and attention to emotion. Cognition and Emotion 26: 119–128. 21500047 10.1080/02699931.2011.559192

[4] de Waal FB (2008) Putting the altruism back into altruism: the evolution of empathy. Annual Reviews Psychology 59: 279–300. 10.1146/annurev.psych.59.103006.093625 17550343

[5] de Wied M, van Boxtel A, Zaalberg R, Goudena PP, Matthys W (2006) Facial EMG responses to dynamic emotional facial expressions in boys with disruptive behavior disorders. Journal of Psychiatric research 40: 112–121. 16176819 10.1016/j.jpsychires.2005.08.003

[6] de Wied M, van Boxtel AV, Posthumus JA, Goudena PP, Matthys W (2009) Facial EMG and heart rate responses to emotion-inducing film clips in boys with disruptive behavior disorders. Psychophysiology 46: 996–1004. 19549069 10.1111/j.1469-8986.2009.00851.x

[7] de Wied M, van Boxtel A, Matthys W (2012) Verbal, Facial and Autonomic Responses to Empathy-Eliciting Film Clips by Disruptive Male Adolescents with High Versus Low Callous-Unemotional Traits. J Abnorm Child Psychol 4: 211–223. 10.1007/s10802-011-9557-8 PMC3267933 21870040

[8] Gill KL, Calkins SD (2003) Do aggressive/destructive toddlers lack concern for others? Behavioral and physiological indicators of empathic responding in 2-year-old children. Developmental Psychopathology 15: 55–71. 10.1017/s095457940300004x 12848435

[9] Feshbach ND, Roe K (1968) Empathy in six- and seven-year-olds. Child Dev 39: 133–145. 5645790

[10] Sterba S, Egger HL, Angold A (2007) Diagnostic specificity and nonspecificity in the dimensions of preschool psychopathology. Journal of child psychology and psychiatry, and allied disciplines 48: 1005–1013. 10.1111/j.1469-7610.2007.01770.x PMC2853244 17915001

[11] Martel MM, Gremillion M, Roberts B, Eye von A, Nigg JT (2010) The structure of childhood disruptive behaviors. Psychological Assessment 22: 816–826. 21133546 10.1037/a0020975 PMC4307591

[12] Williams LM, Hermens DF, Palmer D, Kohn M, Clarke S, et al (2008) Misinterpreting emotional expressions in attention-deficit/hyperactivity disorder: evidence for a neural marker and stimulant effects. Biol Psychiatry 63: 917–926. 18272140 10.1016/j.biopsych.2007.11.022

[13] Pelc K, Kornreich C, Foisy ML, Dan B (2006) Recognition of emotional facial expressions in attention-deficit hyperactivity disorder. Pediatr Neurol 35: 93–97. 16876003 10.1016/j.pediatrneurol.2006.01.014

[14] Sinzig J, Morsch D, Lehmkuhl G (2008) Do hyperactivity, impulsivity and inattention have an impact on the ability of facial affect recognition in children with autism and ADHD? European Child and Adolescent Psychiatry 17: 63–72. 17896119 10.1007/s00787-007-0637-9

[15] Marton I, Wiener J, Rogers M, Moore C, Tannock R (2009) Empathy and social perspective taking in children with Attention-Deficit/Hyperactivity Disorder. J Abnorm Child Psychol 37: 107–118. 18712471 10.1007/s10802-008-9262-4

[16] Dyck MJ, Ferguson K, Shochet IM (2001) Do autism spectrum disorders differ from each other and from non-spectrum disorders on emotion recognition tests? Eur Child Adolesc Psychiatry 10: 105–116. 11469282 10.1007/s007870170033

[17] Braaten EB, Rosen LA (2000) Self-regulation of affect in attention deficit-hyperactivity disorder (ADHD) and non-ADHD boys: differences in empathic responding. J Consult Clin Psychol 68: 313–321. 10780132 10.1037/0022-006X.68.2.313

[18] Yuill N, Lyon J (2007) Selective difficulty in recognising facial expressions of emotion in boys with ADHD. General performance impairments or specific problems in social cognition? Eur Child Adolesc Psychiatry 16: 398–404. 17401608 10.1007/s00787-007-0612-5

[19] Shaffer D, Fisher P, Lucas CP, Dulcan MK, Schwab-Stone ME (2000) NIMH Diagnostic Interview Schedule for Children Version IV (NIMH DISC-IV): description, differences from previous versions, and reliability of some common diagnoses. Journal of the American Academy of Child and Adolescent Psychiatry 39: 28–38. 10638065 10.1097/00004583-200001000-00014

[20] Sattler JM (1992) Assessment of Children (3rd edition). San Diego, CA: Jerome M Sattler Publisher.

[21] Kort W, Schittekatte M, Dekker PH, Verhaeghe P, Compaan EL, et al.. (2005) Wechsler Intelligence Scale for Children-third edition, Dutch version. Psychological Corporation, London.

[22] Achenbach TM, Rescorla LA (2001) Manual for the ASEBA School-Age Forms & Profiles. Burlington, VT: University of Vermont, Research Center for Children, Youth, & Families.

[23] Deschamps PK, Schutte I, Kenemans JL, Matthys W, Schutter DJ (2012) Electromyographic responses to emotional facial expressions in 6–7year olds: A feasibility study. Int J Psychophysiol 85: 195–9. 22634269 10.1016/j.ijpsycho.2012.05.004

[24] Fridlund AJ, Cacioppo JT (1986) Guidelines for human electromyographic research. Psychophysiology 23: 567–589. 3809364 10.1111/j.1469-8986.1986.tb00676.x

[25] Dimberg U, Petterson M (2000) Facial reactions to happy and angry facial expressions: evidence for right hemisphere dominance. Psychophysiology 37: 693–696. 11037045

[26] Larsen JT, Norris JI (2009) A facial electromyographic investigation of affective contrast. Psychophysiology 46: 831–842. 19490516 10.1111/j.1469-8986.2009.00820.x

[27] Eisenberg N, Eggum ND, Di Giunta L (2010) Empathy-related Responding: Associations with Prosocial Behavior, Aggression, and Intergroup Relations. Social Issues and Policy Review 4: 143–180. 21221410 10.1111/j.1751-2409.2010.01020.x PMC3017348

[28] Frick PJ, Loney BR (1999) Outcomes of children and adolescents with oppitional defiant disorder and conduct disorder. In HC Quay & A Hogan (Eds), Handbook of disruptive behavior disorders New York: Plenum: 507–524.

[29] Lahey BB, Loeber R, Hart EL, Frick PJ, Applegate B, et al (1995) Four-year longitudinal study of conduct disorder in boys: patterns and predictors of persistence. J Abnorm Psychol 104: 83–93. 7897057 10.1037/0021-843X.104.1.83

[30] Matthys W, Vanderschuren LJMJ, Schutter DJLG, Lochman JE (2012) Impaired neurocognitive functions affect social learning processes in oppositional defiant disorder and conduct disorder: implications for interventions. Clin Child Fam Psychol Rev 15: 234–246. 22790712 10.1007/s10567-012-0118-7

[31] Matthys W, Vanderschuren LJMJ, Schutter DJLG (2013) The neurobiology of oppositional defiant disorder and conduct disorder: altered functioning in three mental domains. Dev Psychopathol 25: 193–207. 22800761 10.1017/S0954579412000272

[32] Rowe R, Costello EJ, Angold A, Copeland WE, Maughan B (2010) Developmental pathways in oppositional defiant disorder and conduct disorder. J Abnorm Psychol 119: 726–738. 21090876 10.1037/a0020798 PMC3057683

[33] Nock MK, Kazdin AE, Hiripi E, Kessler RC (2007) Lifetime prevalence, correlates, and persistence of oppositional defiant disorder: results from the National Comorbidity Survey Replication. Journal of child psychology and psychiatry, and allied disciplines 48: 703–713. 10.1111/j.1469-7610.2007.01733.x 17593151

[34] Stringaris A, Goodman R (2009) Longitudinal outcome of youth oppositionality: irritable, headstrong, and hurtful behaviors have distinctive predictions. J Am Acad Child Adolesc Psychiatry 48: 404–412. 19318881 10.1097/CHI.0b013e3181984f30

[35] Stringaris A, Goodman R (2009) Three dimensions of oppositionality in youth. J Child Psychol Psychiatry 50: 216–223. 19166573 10.1111/j.1469-7610.2008.01989.x

[36] Dimberg U, Lundquist LO (1990) Gender differences in facial reactions to facial expressions. Biol Psychol 30: 151–159. 2285765 10.1016/0301-0511(90)90024-q

[37] Sonnby-Borgström M, Jonsson P, Svensson O (2008) Gender differences in facial imitation and verbally reported emotional contagion from spontaneous to emotionally regulated processing levels. Scand J Psychol 49: 111–122. 18352980 10.1111/j.1467-9450.2008.00626.x

[38] Newman JP, Curtin JJ, Bertsch JD, Baskin-Sommers AR (2010) Attention moderates the fearlessness of psychopathic offenders. Biol Psychiatry 67: 66–70. 19793581 10.1016/j.biopsych.2009.07.035 PMC2795048

[39] Dadds MR, Perry Y, Hawes DJ, Merz S, Riddell AC, et al (2006) Attention to the eyes and fear-recognition deficits in child psychopathy. British Journal of Psychiatry 189: 280–281. 16946366 10.1192/bjp.bp.105.018150

